# A qualitative study to explore experiences and views of patients and their family members managing steroid‐induced hyperglycaemia (SIH) out of hospital

**DOI:** 10.1111/dme.70138

**Published:** 2025-09-09

**Authors:** Nyangi Gityamwi, Suzanne van Even, Younes Ramazan Younes, Jo Armes, Benjamin C. T. Field

**Affiliations:** ^1^ School of Health Sciences University of Surrey Guildford Surrey UK; ^2^ NIHR Applied Research Collaboration – Kent, Surrey and Sussex, Sussex Partnership NHS Foundation Trust, Sussex Education Centre Hove UK; ^3^ Endocrinology Department, East Surrey Hospital Surrey and Sussex Healthcare NHS Trust Redhill UK; ^4^ Department of Clinical and Experimental Medicine, School of Biosciences University of Surrey Guildford Surrey UK

**Keywords:** diabetes mellitus, hyperglycaemia, primary health care, self management, steroids

## Abstract

**Aim:**

To explore the experiences of patients, families and clinicians managing steroid‐induced hyperglycaemia (SIH) out of the hospital and identify areas for improved care.

**Methods:**

We searched hospital records to identify patients requiring input from the diabetes inpatient team between February 2022 and March 2023 due to steroid usage. Clinicians, patients and their family members were interviewed remotely about their experiences of care and views on how to improve it. Patient characteristics were extracted from hospital records and descriptively summarised. Interview data were subjected to framework analysis.

**Results:**

We interviewed 23 patients (60% male, aged 40–88 years). The median (IQR) glucocorticoid daily dose (prednisolone‐equivalent) was 40 mg (20–60). Fifteen (65%) patients were followed up after discharge by the diabetes specialist team, the remainder being referred to primary care. Nine family members and five diabetes care clinicians were also interviewed.

SIH impacts negatively on patients' and families' physical and social well‐being and increases clinical workload. Participants reported feeling anxious and uncertain when self managing SIH out of hospital, particularly those with multimorbidity and no prior history of diabetes. Regular post‐discharge clinical follow‐up builds patients' confidence and satisfaction, but there was limited post‐discharge follow‐up care, and conflicting advice was provided on SIH management from different care teams. Better discharge care planning, communication, family support and provision of SIH self management resources could improve care and experiences.

**Conclusion:**

Our findings emphasise having robust, individualised, post‐discharge care planning; better communication across care pathways; and provision of skills and resources to all partners in healthcare.

**What's new?**

- Patients and families have limited capacity and confidence to self manage steroid‐induced hyperglycaemia (SIH), and it impacts negatively on their health and social well‐being.
- Patients and families have mixed feelings regarding the quality of care they receive out of the hospital, highlighting limited follow‐up and conflicting information received from different care teams.
- Improving care pathways with robust, individualised written care plans and sharing of skills/training, resources and information between all healthcare partners is warranted.

## 1 BACKGROUND

Despite proven efficacy in a multitude of indications,^1^ glucocorticoids may cause hyperglycaemia^2^ independent of a previous history of diabetes.^1^ It is estimated that the majority of hospital inpatients receiving high‐dose glucocorticoids (≥40 mg/day prednisolone or equivalent) develop steroid‐induced hyperglycaemia (SIH).^3^ If SIH occurs, its onset is usually within the first 48 h after starting high‐dose glucocorticoid therapy.^4^ A prior history of diabetes is associated with a 60%–80% risk of developing SIH during inpatient high‐dose glucocorticoid treatment, compared to 10%–12% in people without prior diabetes.^5^ Most importantly, SIH complicates and prolongs hospital admissions and is associated with increased risks of cardiovascular events, infections and mortality.^6^

While management of SIH might be relatively straightforward in the acute clinical setting, the situation is more difficult after discharge from hospital, particularly if this occurs with uncorrected hyperglycaemia or when glucocorticoids are to be continued. Self managing SIH at home often requires frequent self monitoring of blood glucose and adjustments to diet, lifestyle and medications. This can be challenging as dose requirements for insulin may change frequently following steroid dosage adjustment.^7^ Incorrect insulin dose adjustment may lead to disabling or life‐threatening hypoglycaemia. Anecdotal discussions suggest that managing SIH at home can cause distress, anxiety and risk of adverse events. Nevertheless, there is limited literature on experiences of managing SIH out of hospital, and it is unclear how the condition impacts everyday life. This study aimed to explore (1) management and care practices of SIH and the perceived quality, (2) personal experiences and impact of SIH and (3) considerations to improve care and experiences.

## 2 METHODS

### 2.1 Study design

We conducted in‐depth interviews with patients and their family members and clinicians. We also retrieved demographic and treatment data from patients' inpatient care records.

### 2.2 Study setting

The study was conducted at East Surrey Hospital in Redhill, a large district general hospital managed by Surrey and Sussex Healthcare NHS Trust. The hospital has around 700 beds and serves a catchment population of approximately 740,000 people across east Surrey and north‐east West Sussex.

The inpatient diabetes team consists of two full‐time Diabetes Specialist Nurses (DSN) posts, filled on rotation from within the department's cohort of eight DSNs, supported by a consultant diabetologist. A networked blood glucose monitoring system is used to identify patients with dysglycaemia, and the team also receives direct referrals from other inpatient teams. The clinical opinion of the team is entered systematically in a dedicated electronic patient record template, allowing searches to be performed easily. Between 02/2022 and 03/2023 (study period), the inpatient diabetes team recorded a total of 3796 consultations, of which 7% (*n* = 268) were for SIH. The hospital follows the Joint British Diabetes Societies (JBDS) guideline on inpatient care of steroid‐induced hyperglycaemia.^8^

### 2.3 Participants and recruitment

**FIGURE 1:**
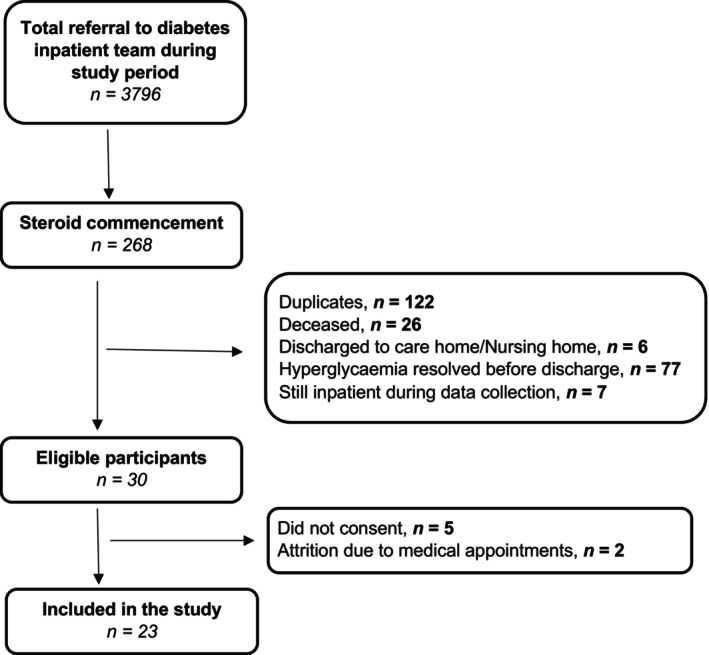
A flow diagram showing the number of patients screened against eligibility criteria, those excluded and the final number of participants included in the study.

We identified potential patient participants through searches of inpatient electronic care records. Purposive sampling was employed to recruit participants in the study. Eligibility criteria comprised: adults aged 18 years and above; who have received steroid treatment while admitted; and who required input from the diabetes inpatient team after hospital discharge between February 2022 and March 2023. We only included individuals who were able to speak English. Written informed consent was a prerequisite for participation. Figure 1 shows the number of patients screened and selected for inclusion in the study. Those who consented were recruited to the study and asked to share the study information and invitation with family members involved with their care. Consented patient participants invited their relatives to take part. We recruited family members involved in directly supporting the participants. Family members were also recruited after giving written consent. Clinician participants were approached directly by members of the study team and were recruited after giving written consent.

### 2.4 Data collection

Interviews were conducted via telephone or Zoom, depending on participants' preference, for 30–40 min, by authors experienced with interviews, NG (PhD, Research Fellow) and SvE (MSc, Research Assistant), both females. There was no established relationship between interviewers and participants before the study, and only participants and the researcher were present during interviews. A semistructured interview guide was developed based on the research questions. This was not pilot tested, but follow‐up questions and probes were used to further explore the topic depending on participants' responses.^9^ Data saturation was discussed and agreed upon by authors NG and SvE. With participants' consent, all interviews were either video or audio recorded to aid data transcription. Interviews conducted via Zoom were automatically transcribed using the built‐in transcription functionality and then checked and pseudonymised by the research team. For telephone interviews, audio recordings were pseudonymised and then securely sent to the approved third party for verbatim transcription. Transcripts were not returned to participants for comments, but feedback on findings was gained through an online workshop. Clinical data were extracted from electronic patient records.

### 2.5 Ethical considerations

Ethical approval was granted by the NHS Health Research Authority (Reference: 22/SC/0263).

### 2.6 Data analysis

Interview data were analysed using Framework Analysis as described by Ritchie and Spencer.^10^ Transcripts were checked by two authors (NG and SvE), who then developed a coding framework. Two authors (NG and SvE) coded the data into an MS Excel spreadsheet and developed themes, which were discussed and final themes agreed upon by all authors.

## 3 RESULTS

### 3.1 Participants

Of 30 identified eligible patients, 25 consented to participate in the study and 23 were interviewed (Figure 1). Reasons for refusal (*n* = 5) or drop out (*n* = 2) were mainly due to being unwell or having a hospital appointment. Participants were predominantly men (60%), with a median age of 68 (range 40–88) years. Table 1 presents the baseline characteristics of the interviewed participants, including blood glucose levels, diabetes medication and steroid usage. Nine family members consented and were included in the study (interviewed individually [*n* = 5] or jointly with the patient participant [*n* = 4]). We also interviewed 5 clinicians (3 diabetes specialist nurses [DSNs], 2 GPs).

**TABLE 1:** Background and characteristics of patients' participants included in the study (*n* = 23), diabetes status and medication used before and after glucocorticoid exposure.

Study ID	Diabetes type	Pre‐admission diabetes medication	Latest HbA_1c_ prior to steroids mmol/mol (DCCT%)	Intervals between the last HbA_1c_ and steroid treatment (days)	Indications for steroids	Steroid	Steroid dose (mg/day)	Inpatient diabetes medication	Blood glucose level at discharge	
									Blood glucose range (mmol/mol)	HbA_1c_ mmol/mol (%)
PP2	2	Metformin, DPP4i	55 (7.2%)	119	Vasculitis	Prednisolone	50	SU, basal insulin	9–24	
PP3	Steroid‐induced	N/A	46 (6.4%)	32	Vasculitis	Prednisolone	40	SU		46 (6.4%)
PP4	2	Rapid & basal insulins	88 (10.2%)	56	Haematological malignancy	Dexamethasone	20	Rapid and basal insulins	6–22	
PP5	2	DPP4i	46 (6.4%)	171	Autoimmune multi‐system disorder	Prednisolone	2.5	SU, metformin		136 (14.6%)
PP6	2	Metformin, SU, SGLT2i	58 (7.5%)	67	Haematological malignancy	Prednisolone	100	Basal insulin		65 (8.1%)
PP9	2	Metformin	42 (6.0%)	83	Benign haematological disease	Prednisolone	80	Rapid and basal insulins	8–22	
PP10	2	Metformin	45 (6.3%)	269	Infective exacerbation of chronic respiratory disease	Prednisolone	10	Rapid and basal insulins	7–18	
PP11	2	Metformin, SGLT2i	57 (7.4%)	46	Inflammatory bowel disease	Prednisolone	35	SU, basal insulin	3–27	
PP13	Steroid‐induced	N/A	46 (6.4%)	73	Solid organ transplant recipient	Prednisolone	2.5	SU		50 (6.7%)
PP14	2	Metformin, DPP4i	65 (8.1%)	68	Infective exacerbation of chronic respiratory disease	Prednisolone	15	Premixed insulin		66 (8.2%)
PP15	2	Metformin	67 (8.3%)	117	Viral pneumonia	Dexamethasone	6	SU		66 (8.2%)
PP16	2	Metformin, DPP4i	109 (12.1%)	7	Autoimmune connective tissue disease	Prednisolone	60	Premixed insulin		112 (12.4%)
PP17	2	Metformin, SU	NIL	NIL	Haematological malignancy	Prednisolone	40	SU	5–17	
PP18	2	GLP1RA, premixed insulin	75 (9.0%)	13	Autoimmune dermatological disease	Prednisolone	30	Rapid insulin, premixed insulin	8–20	
PP19	Steroid‐induced	N/A	49 (6.6%)	4	Inflammatory bowel disease	Prednisolone	40	SU		49 (6.6%)
PP20	2	Metformin, DPP4i	48 (6.5%)	72	Autoimmune digestive disease	Prednisolone	20	Basal insulin		50 (6.7%)
PP21	2	GLP1RA	41 (5.9%)	0	Infective exacerbation of chronic respiratory disease	Prednisolone	10	SU	10–18	
PP22	2	Metformin, GLP1RA, premixed insulin	73 (8.8%)	133	Haematological malignancy	Prednisolone	100	Premixed insulin		111 (12.3%)
PP23	Steroid‐induced	N/A	36 (5.4%)	59	Inflammatory bowel disease	Prednisolone	60	Rapid and basal insulins		49 (6.6%)
PP24	2	GLP1RA, premixed insulin	109 (12.1%)	9	Infective exacerbation of chronic respiratory disease	Prednisolone	30	Premixed insulin		75 (9.0%)
PP25	2	Metformin, basal insulin	68 (8.4%)	73	Autoimmune digestive disease	Prednisolone	30	Rapid and basal insulins	8–20	
PP26	2	Metformin	54 (7.1%)	118	Autoimmune neurological disease	Prednisolone	85	SU		54 (7.1%)
PP27	2	Metformin, SU, SGLT2i	61 (7.7%)	33	Non‐infective exacerbation of chronic respiratory disease	Prednisolone	35	SU		61 (7.7%)

*Note*: Basal insulin = intermediate‐ or long‐acting insulin analogue or human‐sequence neutral protamine Hagedorn insulin preparation.

Abbreviations: DPP4i, dipeptidyl peptidase‐4 inhibitor; GLP1RA, glucagon‐like peptide‐1 receptor agonist; SGLT2i, sodium‐glucose co‐transporter‐2 inhibitor; SU, sulfonylurea; Rapid insulin, rapid‐acting insulin analogue.

### 3.2 Inpatient care records

**FIGURE 2:**
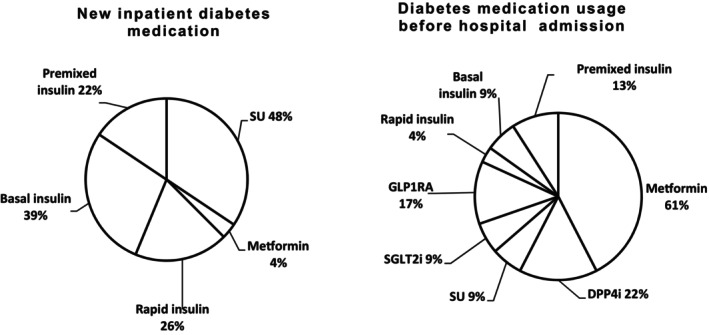
Proportion of patient participants and the type of medication used before and after hospital admission (*n* = 23). Basal insulin = intermediate‐ or long‐acting insulin analogue or human‐sequence neutral protamine Hagedorn insulin preparation; DPP4i, dipeptidyl peptidase‐4 inhibitor; GLP1RA, glucagon‐like peptide‐1 receptor agonist; SU, sulfonylurea; SGLT2i, sodium‐glucose co‐transporter‐2 inhibitors; Rapid insulin, rapid‐acting insulin analogue.

Twenty (86%) patient participants had pre‐existing diabetes. Diabetes medication used by each participant before and during admission is described in Table 1. Figure 2 presents the proportion of participants using the reported medications before and during admission (i.e. new inpatient prescriptions).

The median (IQR) glucocorticoid daily dose (prednisolone‐equivalent) was 40 mg (20‐60 mg), for respiratory, rheumatological, gastrointestinal or haematological indications. Fifteen (65%) patients were followed up after discharge by the diabetes specialist team; the remainder were referred to primary care.

### 3.3 Interviews with patients and their family members

**FIGURE 3:**
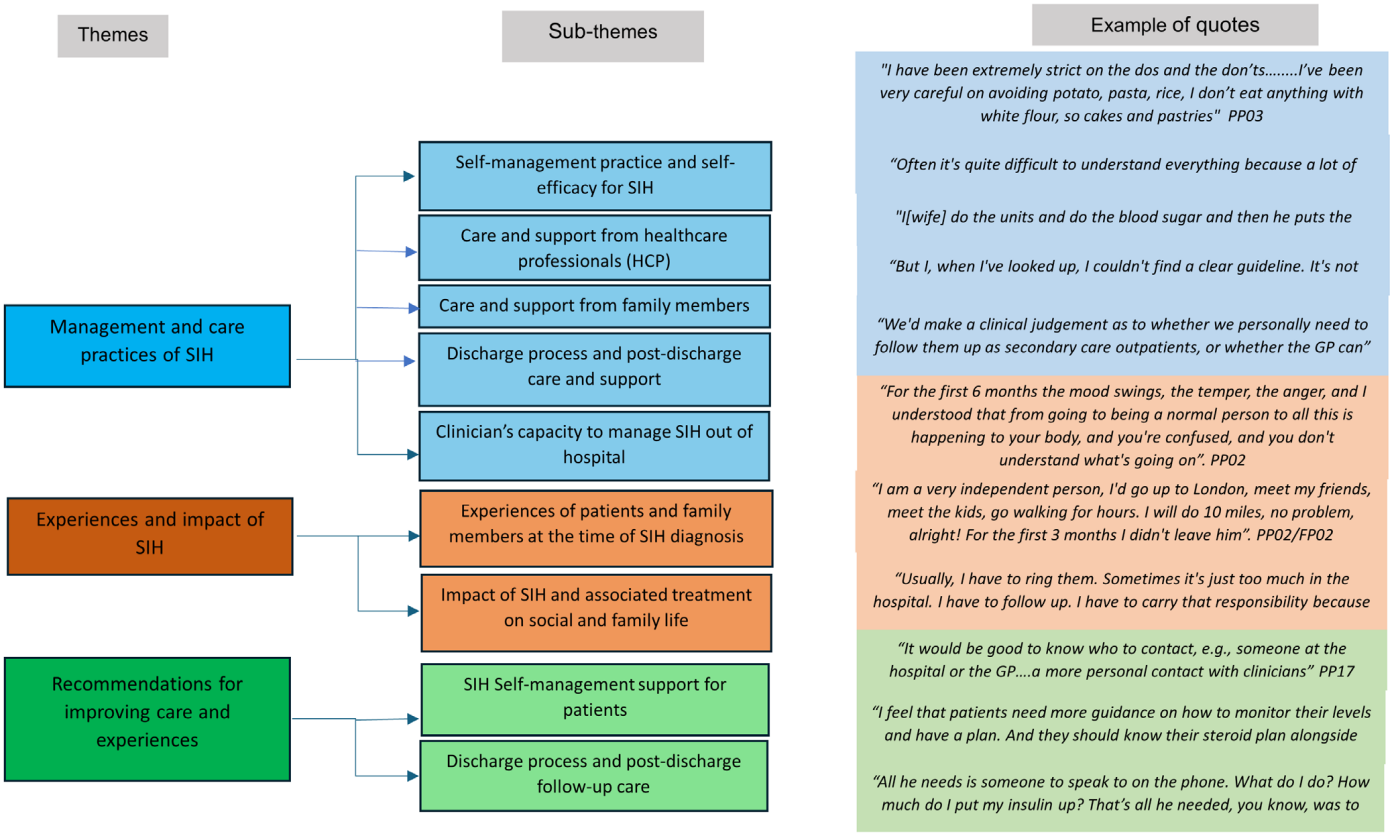
Code tree illustrating major themes and sub‐themes developed with an example of a quote from each theme.

Data from patients and family interviews were analysed and summarised into three major themes according to the research questions: (i) Management and care practices of SIH, (ii) Experiences and impact of SIH, (iii) Recommendations for improving care and experiences. Figure 3 presents a coding tree illustrating themes and sub‐themes developed.

#### 3.3.1 Theme 1: Management and care practices of SIH

##### Sub‐theme (i): Self management practices and self‐efficacy for SIH

Both dietary and pharmacological approaches are used in managing SIH (Table 2, PP03). Participants with a long history of diabetes or a health education background demonstrated good awareness of their treatment as they were able to describe their treatment and self‐medicate without or with little support (Table 2, PP13). Others reported struggling to manage SIH, requiring continuous close support. For those who had not used insulin before, learning to both inject insulin and monitor their blood glucose levels was a new experience, and they reported finding it somewhat challenging. There was also a concern regarding the occurrence of hypoglycaemia when using insulin (Table 2, PP17).

**TABLE 2:** Quotes from study participants describing care and management practices for SIH.

**Sub‐theme (i): Self management practices and self‐efficacy for SIH**
*‘I have been extremely strict on the dos and the don'ts… I've been very careful on avoiding potato, pasta, rice, I don't eat anything with white flour, so cakes and pastries’. PP03*
*‘I've had a long‐term condition in my life, so you know I'm pretty good at managing it. I'm pretty resilient, really resilient, and to be perfectly honest, I don't really want that much input, because I'm sick of you know hospitals’. PP13*
*‘I just had this [diabetes] for about 14 years, 12–14 years, and initially I was managing it myself, but eventually I couldn't….it was fully under control before, … but every time I have this sort of 2 more days of steroids, it's a bit all over the place, and then it kind of settles itself again’. PP17*
**Sub‐theme (ii): Care and support from healthcare professionals (HCP)**
*‘I had a lot of guidance from the pharmacist. They've been excellent, actually’. PP03*
*‘Can't fault the medical, the doctors, the nurses, everybody, you know, was absolutely amazing. The diabetic specialist came in and spoke to me, and I spent more than an hour with the nurse going through the medication’. PP02*
*‘Often it's quite difficult to understand everything because a lot of conversation is in medical terms, isn't it?’ PP03*
*‘I think, I was left to manage it, to make the decisions, but obviously not knowing what those symptoms look like or feel like and when it got to that stage it was too late. Being on the Gliclazide, then realising that I shouldn't be taking it or taking half. Then find that I didn't actually really need it. So, it's just managing it bit by bit, but I know it's trial and error’. PP05*
**Sub‐theme (iii): Care and support from family members**
*‘So I would make sure that she had, like food available with sugar content that she could access and all those, make sure she was topped up, and making sure that she was kind of taking the relevant medication when she needed, and make sure that she had all her prescriptions update’. PP05*
*‘I work with a girl who was, you know, a diabetic nurse specialist as well like oh, sometimes, you know, I text her and say, Julie, I've got this upset tummy, what should I do?’ PP13*
*‘I[wife] do the units and do the blood sugar and then he puts the injection in himself’. PP04*
*‘Well, my husband's been feeding me because he's had to do all the cooking, so… so my diet is probably not as good as it should be at the moment’. PP27*
*‘My wife is with me all the way, and she has a little knowledge. She works in the hospital, as well, so she had a little knowledge, of what's going on’. PP22*

*Note*: PP denotes patient participants; FP denotes family member participants.

##### Sub‐theme (ii): Care and support from healthcare professionals (HCP)

Participants reported receiving follow‐up care from hospital diabetes specialist nurses in the form of phone calls either once or twice a week, depending on the individual's support needs. This brought a sense of confidence and reassurance. Participants also reported receiving a ‘*discharge note*’ and leaflets with treatment and management instructions at the point of discharge, which they found somewhat helpful. Those on insulin were shown how to inject insulin and given finger‐prick tests to self monitor blood levels at home. One participant mentioned receiving guidance from a dietitian regarding diet, while others received guidance on medications from pharmacists (Table 2, PP02, PP03). Nevertheless, participants had mixed opinions and satisfaction with the level of support and care they received from health professionals. While most continued to be cared for as outpatients by the hospital diabetes team and expressed great satisfaction, there were a few concerns, particularly among those discharged to community/primary care. They were concerned about the lack of regular check‐ups with healthcare professionals and not being able to get a GP appointment and felt ‘left out. They were unsure how to self manage their condition or were given conflicting advice by different care teams.

There was also a feeling of being ‘ignored’ or not being involved enough in their care when clinicians did not explain changes in medications or use medical jargon (Table 2, PP03). Others were not sure who to go and see (among multiple care teams they see) when concerned about their blood glucose levels (Table 2, PP05). In particular, one participant described *‘feeling abandoned’* as they never had the chance to talk to anyone specifically about SIH. Another reported finding was that this was distressing to the point of becoming suicidal. One participant reported not receiving a timely medication(s) review.

##### Sub‐theme (iii): Care and support from family members

Most participants described having close family members who offered different levels of support depending on the needs of the patient. Some described themselves as being highly dependent on relatives as they needed support attending clinic appointments, administering medication, including insulin injections and personal care due to being unwell. For others, minimal support such as prompting about medication, searching for information online or assisting with domestic chores was described (Table 2, PP05, PP04). The interviews suggested that patients also sought or received support from the wider community outside their family, with one participant reaching out to a work colleague for support (Table 2, PP13). Nevertheless, not everyone appeared confident in the capacity of their family members to provide much‐needed support. For example, one participant described her concerns about the quality of her diet (Table 2, PP27). Two participants had family members who were health professionals, and so, they felt confident and reassured by their support system (Table 2, PP22).

#### 3.3.2 Theme 2: Experiences and impact of SIH on patients and their families

##### Sub‐theme (i): Experiences of patients and family members at the time of SIH diagnosis

Participants described the events and incidents at the time of SIH diagnosis as ‘*shocking’* and *‘awful*’. They described feeling unusually ill and, in most cases, were unsure what caused the changes they were experiencing in their health and well‐being, which made them anxious and stressed. This was independent of participants' history of diabetes, as those with pre‐existing diabetes equally described their blood glucose levels being out of control after they were initiated on steroid therapy (Table 3, PP02). For participants with multimorbidities, this was doubly burdensome as some were unsure as to whether their experiences were due to steroids or other health conditions (Table 3, PP14). The reduced contacts during COVID‐19 were perceived as exacerbating the fear, as they could not reach their clinicians as they used to (Table 3, FP04). When family members were asked about their experiences, they described sharing a sense of worry and not being sure of the changes they observed in their loved ones (Table 3, FP20).

**TABLE 3:** Quotes from study participants describing experiences and impact of SIH on patients and their family members.

**Sub‐theme (i): Experiences of patients and family members at the time of SIH diagnosis**
*‘I was in a bit of a panic, because obviously there was limited visiting at that time and then because of Covid, no visiting’. FP04*
*‘We'd planned to go out somewhere. And her vision kind of, like, started going really… really fuzzy. I think this was one of the first times that I'd experienced it whilst I was actually with her for a long time. So, her vision was going really funny, and she was starting to feel really sick. We didn't have, like, a sick bag or anything in the car, so I was, like, getting a bit worried and things’. FP20*
*‘From going to being a normal person to all this is happening to your body, and you're confused, and you don't understand what's going on’. PP02*
*‘I think it's a blinking nuisance because there are two major things [diabetes and COPD], aren't they? I think they're both a nuisance’. PP14*
*‘For the first 6 months the mood swings, the temper, the anger, and I understood that from going to being a normal person to all this is happening to your body, and you're confused, and you don't understand what's going on’. PP02*
*‘Well, I just couldn't control my body waste at all, it just left me, as soon as I ate, you know, it was out the other end, and they put me on the slow release one and that was just as bad, and that was really to bring down my sugar levels, which it did, but that was an awful, embarrassing time, because I couldn't really leave the house’. PP03*
*‘They put me on hydroxychloroquine which had an effect, I was being sick. And now more methotrexate which my body's again, I've just had a little one…. I've been off sick at the moment, I was just throwing up and sick and lost weight again’. PP05*
**Sub‐theme (ii): Impact of SIH and associated treatment on social and family life**
*‘It was just the worry of not being able to get in touch with her every second of every day, to make sure that she was okay…. And then, nothing, no kind of information leaflets or any guidance and support for someone witnessing someone go through that, and how you can help that person really’. FP01*
*‘But the impact for me was far greater because it's quite scary. I couldn't even drive, and to watch TV I had to wear different glasses……….and not wanting to go out, because I just don't feel comfortable in my surroundings’. PP05*
*‘We would have gone for 3 or 4 months [on holiday]. Now it's 3 weeks. The last time we went was 2 weeks, because of all the tests that he has to do, and all of that, you know, and before we go, we get permission’ PP02/FP05*
*‘I am a very independent person, I'd go up to London, meet my friends, meet the kids, go walking for hours. I will do 10 miles, no problem, alright! For the first 3 months I didn't leave him’. PP02/FP02*
*‘My diet… everything is changed, and I eat healthy meals. II tell my family not to eat too much sweet. So everyone is eating smaller portions and very healthy’. PP06*

*Note*: PP denotes patient participants; FP denotes family member participants.

##### Sub‐theme (ii): Impact of SIH and associated treatment on social and family life

Participants reported that their confidence in going out to social events was reduced due to the need to do the finger‐prick test frequently. Poor vision was also reported, impacting their driving ability, independence and social life. For those who required more support, the sense of being dependent or feeling like a burden to the family caused distress. Difficulties in focusing and articulating things were also reported, and this impacted their ability to work (Table 3, PP05).

The family members shared a sense of distress and anxiety when supporting the care of their loved ones. Some felt worried, unsure whether they were doing things right (particularly if there is multimorbidity), whereas others felt that they did not receive enough support (Table 3, FP01). Not knowing what was going to happen and when made them feel they needed to be around all the time, impacting their personal social life. Families could no longer go on holiday as they used to due to frequent hospital appointments and treatment (Table 3, PP02/FP05). Nevertheless, some families reported that their involvement in care for SIH helped them to have a better understanding of diabetes.

#### 3.3.3 Theme 3: Recommendations for improving care and experiences managing SIH

##### Sub‐theme (i): Discharge process and post‐discharge follow‐up care

Participants indicated the need for frequent follow‐up, particularly immediately following discharge, to ensure that they were adapting well and could self manage. They also suggested that post‐discharge care should be personalised and ensure they have easy access to GPs (Table 4, PP02). Having a point of contact whom they could call when they have concerns or queries specific to SIH was thought to be helpful (Table 4, PP17), as was having someone who could give personalised information and advice rather than generic advice, considering that the majority of patients had other underlying health conditions (Table 4, PP09). The interviews highlighted the need to improve the prescription service to ensure availability or access to the blood glucose test kits when needed (Table 4, FP01). It was also recommended that clinicians involve patients and their family members, where relevant, in discussions about their treatment plan so that they can better support their loved ones (Table 4, PP05).

**TABLE 4:** Quotes from study participants describing recommendations to improve care and experiences managing SIH out of hospital.

**Sub‐theme (i): Discharge process and post‐discharge follow‐up care**
*‘Somebody could have come in within the first week, and see how we've settled in. See how I am doing, because they have a list like 15 different medications, you know. If I was comfortable with it. If I was administering, it properly’. PP02*
*‘It would be good to know who to contact*, e.g., *someone at the hospital or the GP….a more personal contact with clinicians’. PP17*
*‘It's the GPs, and just… you know, to get a prescription I would go, put a prescription in, it wouldn't be right at the chemist, I'd go back to the doctor's, they wouldn't do it right again, I'd go back to the chemist, so I could take six trips to get one prescription’. FP01*
**Sub‐theme (ii): SIH self management support for patients**
*‘I'll look at them online and look at those drugs regularly, just to keep myself refreshed on what I can do and what I can't do’. PP27*
*‘I think as long as the technology used is designed to be as user‐friendly as possible, considering the people that would be using it, for example, having text to speech if their vision is impaired, it would make things slightly easier’. FP02*
*‘All he needs is someone to speak to on the phone. What do I do? How much do I put my insulin up? That's all he needed, you know, was to have someone give them the okay to put the insulin up’. FP05*
*‘It's almost like talk to my daughter, because she'll understand it better, or she's going to relate to me when I'm bit more with it. Cause at the moment you tell me, I'm just going “Yeah, yeah, yeah,” nodding my head. [laughs]. I'm just so agreeing, but not properly understanding, the reasoning that makes sense, and probably that would be more helpful. relating to another family member or somebody. Just so they heard it, in case I've mis‐heard it, or only taken bits of the information in’. PP05*
*‘I needed someone to say this is what's happening, this is what we're going to do about it, this is what you need. I could have spoken to somebody about all the insulin and about the steroids, somebody that could listen and somebody could do something to review me and I had a care plan’. PP09*

*Note*: PP denotes patient participants; FP denotes family member participants.

##### Sub‐theme (ii): SIH self management support for patients

Participants called for measures to be taken to ensure that patients receive sufficient and correct information in an accessible way (lay language) about their treatment and self management of SIH. This should include any changes in medications and information about the signs and symptoms of high and low blood glucose so that they can recognise this early when it happens (Table 4, PP09, FP05).

Participants also suggested that, if well designed and provided, technology could help with SIH self management, in particular through aiding consultations with care professionals, information on signs and symptoms, and even glucose monitoring devices (Table 4, FP02). Nevertheless, the need to have a human on the other side of technology to help patients respond to the changes picked up by the technology was highlighted (Table 4, FP05).

### 3.4 Interviews with clinicians

The hospital diabetes specialist nurses and GPs also provided their perspectives on the SIH management practices, experiences and impact of SIH, and recommendations for improving care services. These are presented in three themes as follows:

#### 3.4.1 Theme 1: Management and care practices of SIH

##### Sub‐theme (i): Discharge process and post‐discharge care and support

It was reported that, at the point of discharge, clinical judgement is used to decide whether a patient needs follow‐up care as an outpatient instead of with the GP/practice nurses (Table 5, CP06). Thirty‐five per cent (*n* = 8) of the patient participants in our study were discharged to the GP. When the GPs were asked about the discharge process, they explained that they usually get a letter from a consultant informing them about the patient's diagnosis of SIH. There were also cases where individuals consulted the GP themselves to report their diagnosis after being advised by their respective clinics (Table 5, CP02).

**TABLE 5:** Quotes from clinician study participants describing the care practices and experiences managing SIH out of hospital.

**Sub‐theme (i): Discharge process and post‐discharge care and support**
*‘We'd make a clinical judgement as to whether we personally need to follow them up as secondary care outpatients, or whether the GP can’. CP06*
*‘I would suggest that they follow up with the GP. I personally wouldn't follow that up, and I would hand that over to the GP depending on the treatment…. But I do have some colleagues who would continue to call the patients after discharge to see how their levels are. But that's a bit of a grey area. Because they don't necessarily need to be referred to secondary care’. CP06*
*‘They're contacting us, saying he's not stable, but this isn't our plan. So, then we're trying to say, well, speak to the hospital diabetes nurse specialist and then the hospital diabetes nurse specialist is thinking this man is in the community, what has it got to do with me? it's just really difficult’. CP07*
*‘The standard what happens is we get a letter from the consultant who's looking after the patient, whether it's a rheumatologist or the oncologist saying that your patient has now been diagnosed with diabetes, and because they've got this, what they call a steroid‐induced diabetes. Sometimes patients come to us because they've been told in the clinic, and they come and see us. And now we're here and what should we do?’ CP02*
*‘We code them as steroid‐induced diabetes, and they get on, our diabetes register. We would do with them what we do with every person who's diagnosed with diabetes…. We call everyone… We do their bloods’. CP02*
*‘I give them telephone calls while they're home and guide them too and give them a number that they can also call if they have any worries’. CP04*
*‘Patients are given a leaflet on steroids, including information about how to treat SIH*, e.g., *how often they need to monitor their blood glucose levels, what they need ot do if they stop steroids/lower their dose and titrating down tablets and/or insulin to manage SIH. The leaflet includes information on what to do if glucose levels go above 12 and who to contact (local GP and ask DN in hospital for advice)’. CP05*
**Sub‐theme (ii): Clinician's capacity to manage SIH**
*‘We don't have a Trust‐specific protocol, but we do base all our practice off the JBDS guidance’ CP06*
*‘But I, when I've looked up, I couldn't find a clear guideline. It's not clear to me, and that may be lack of my understanding’. CP02*
*‘So, they're bound to following instruction charts and the instruction charts that are inflexible; they're not diabetes trained, they're just going in and administering…They're trained to administer insulin, but they're not trained to titrate according to blood glucose, according to dietary intake. They're not allowed to modify the prescribed doses’. CP07*
*‘And any changes we make, take a couple of days to filter through, cause they've gotta be prescribed on the charts. Then they've got to be emailed to the nurses. Then they've got to be uploaded onto the patient's care plan at the end. And all of this. It takes about 48 h to implement’. CP07*
*‘I've had extra training in managing diabetes. I'm really pretty confident. My lead nurse, who has been there the same length of time as I have and has done lots of extra diabetes training. She's pretty confident’. CP07*
*‘The power of practice lies in what we don't prescribe, so I feel that sometimes… that's the challenge for me personally, it's not a patient challenge, it's a clinician challenge’. CP02*
*‘Usually, I have to ring them. Sometimes it's just too much in the hospital. I have to follow up. I have to carry that responsibility because sometimes patient finds it difficult to get in touch with GPs’. CP04*
*‘Filling in community administration forms and then scanning them on, emailing them off, making sure they've been received at the other end, all of that's quite labour‐intensive and time consuming…’. CP07*

*Note*: CP denotes clinician participant.

Regarding post‐discharge care and support, the interviews with DSNs suggested that the inpatient diabetes care team was not mandated to provide follow‐up care as it was expected that the care responsibility would be picked up by the GP or community care services (Table 5, CP06). Nevertheless, one DSN reported providing personalised advice and, even though patients are usually advised to follow‐up with their GP, offered to stay in regular contact with a patient after discharge (Table 5, CP04).

The interviews showed that it is standard practice to provide information leaflets to patients at the point of discharge, summarising instructions on how to self manage SIH at home. This included how often they need to monitor their blood glucose levels, the target levels and what to do if glucose levels go out of the range. The leaflets also explained about dosage, when to take what medication (and when to stop taking medication(s)), as well as the medications' side effects (Table 5, CP05).

The GPs also described the care they provide to patients discharged to their practices, which included regular follow‐ups (monthly check‐ups) and provision of glucometers to patients. One GP described offering patients the option of contacting her as necessary. Furthermore, community nurses did home visits to help administer insulin to frail elderly patients who were unable to do it themselves and lacked family support. However, GPs also reported that since most patients have underlying conditions, they usually receive the needed care and support from other services (e.g. Macmillan for cancer patients) (Table 5, CP02).

The interviews suggested that there is inefficient communication between secondary and primary care during the discharge process, rendering it difficult for primary care to plan and deliver post‐discharge care. It was also explained that medication review by the GP/DSNs might take a few days to filter through to community nurses, delaying the delivery of appropriate treatment (Table 5, CP07). The interviews also revealed that the care pathways for SIH are not clear, particularly for patients with continuing steroid treatment and co‐morbidities.

##### Sub‐theme (ii): Clinician's capacity to manage SIH out of hospital

The DSNs reported using the Joint British Diabetes Society guidelines to care for patients who were managed for SIH as outpatients (Table 5, CP02). On the contrary, GPs reported following the treatment plan that was initiated in the hospital and rarely titrating the dose (Table 5, CP07).

Although interviewed GPs reported being comfortable about initiating and titrating insulin, it was revealed that newly qualified GPs and nurses did not have specialised training and therefore lacked confidence in managing SIH and diabetes in general. GPs explained that they found it difficult to manage patients who were more prone to glucose variability and those who were insulin resistant. The GPs also expressed feeling less confident in managing SIH because it was very dependent on steroid administration, over which they had no control or involvement. They also found it challenging when patients were discharged to primary care with a treatment regimen that the GP did not agree with (Table 5, CP02, CP07).

Clinicians highlighted the impact SIH had on their workload as care for SIH is usually unscheduled and sometimes urgent, unlike T2D or T1D, where care could be preplanned/scheduled. This was exacerbated by the administrative work associated with managing SIH (Table 5, CP07). From the DSNs' perspective, SIH added to their workload too when they had to follow‐up on patients after hospital discharge. One participant explained that they might speak with patients twice a week, based on how well the patient was titrating the insulin (Table 5, CP04).

#### 3.4.2 Theme 2: Clinicians' recommendations for improving care

##### Sub‐theme (i): Improving discharge process and post‐discharge follow‐up care

Clinicians called for better discharge processes with care planning ahead of discharge and personalised discharge care plans. The care plan should be discussed and communicated with primary and community care to help ensure continuity of care, as any member of the community team would be able to take responsibility for the care and meet care/clinical expectations (Table 6, CP07).

**TABLE 6:** Quotes from clinician study participants describing their recommendations for improved care.

**Sub‐theme (i): Improving discharge process and post‐discharge follow‐up care**
*‘I think that that's better communication with secondary care would be helpful. Cause that it's always quite time consuming and difficult to get hold of colleagues’. CP07*
*‘Liaising with primary care team is key part—GP should monitor blood glucose levels patients, provide patients with glucose level strips’. CP05*
*‘I mean when it comes to insulin, and people really don't wanna take their responsibility if they're not a specialist’. CP04*
*‘I would, I would say in our area, the community nurses definitely need some enhanced insulin and diabetes training and skills’. CP07*
*‘For me an ideal outcome would be if they could be clear guidelines there aren't any guidelines when somebody gets diagnosed with steroid‐induced diabetes for a normal diabetes person’. CP02*
**Sub‐theme (ii): Improving SIH self management support for patients**
*‘I feel that patients need more guidance on how to monitor their levels and have a plan. And they should know their steroid plan alongside their plan for monitoring their glucose levels’. CP06*
*‘Most people don't need a lot of extra support to be very honest, but it's that confidence is that fear, when I need help, will there be someone to go to?’ CP02*
*‘For patients to be provided with information on how steroid treatment could have an impact on their blood glucose levels, patients need to be reassured that SInHG is only for a short time (for the duration that they have to be on steroids)’. CP04*

*Note*: CP denotes clinician participant.

The interviewed GPs and DSNs described the need for enhanced diabetes care training for GPs, practice and community nurses to improve their skills, confidence and capacity to pick up responsibility for the care of people with SIH after hospital discharge (Table 6, CP07). They also called for funded training in the use of technology so that GPs can initiate continuous blood glucose monitoring, as currently it is only initiated in secondary care (Table 6, CP02).

##### Sub‐theme (ii): Improving SIH self management support for patients

Clinicians explained that patients could benefit from having access to clear information on their steroid treatment plan alongside guidance about monitoring their glucose levels (Table 5, CP06). Having a hotline specifically for blood glucose‐related problems was also thought to be beneficial (Table 5, CP07).

## 4 DISCUSSION

The findings from our in‐depth interviews highlight the worries, concerns and adverse events experienced by both patients and families in managing SIH, regardless of a previous history of diabetes, suggesting that they were under‐prepared to manage SIH. This calls for more effort to be placed on patient education to improve medication awareness in line with the SIH management consensus.^8^ In the present study, patients and family members expressed greater satisfaction where there was a good understanding of medications used, emphasising the importance of improving medication awareness for patients.^11^

In terms of managing SIH, patient participants reported using both lifestyle modification and pharmacological approaches (oral medications and insulin) in line with the available recommendations.^12^ However, there were mixed feelings about self‐efficacy to self manage SIH, particularly with insulin. A previous history of diabetes and treatment with insulin helped with confidence in managing SIH, perhaps due to prior knowledge, skills and lived experience^13^ in monitoring blood glucose levels and self‐administering insulin.^14^ Ongoing educational interventions may improve patients' overall confidence to self manage SIH, improving their experiences and glycaemic control.^15^ Indeed, when asked how care could be improved, participants highlighted the need for accessible SIH‐specific information resources coupled with technologies to aid glucose monitoring. This will potentially help to improve self‐efficacy for diabetes self management, satisfaction and reduce psychological distress.^16^

It was also evident that people with co‐morbidities had more difficulties self managing SIH at home as they described struggling to cope with multiple symptoms, complex treatment regimens and inconsistent information they sometimes received from different care teams.^17^ Inconsistent information has been shown to compromise diabetes self management^18^ as patients are likely to be confused, anxious and less confident, which may affect adherence to treatment.^19^ This calls for the implementation of coordinated multidisciplinary working to support the continuity of care for patients.^20^, ^21^

Our interviews with GPs and DSNs suggested that there is limited clinical capacity within primary care to take on care responsibility for SIH, calling for the provision of enhanced diabetes and SIH management training and shared learning between care teams.^22^ This might have been exacerbated by the reported inefficient communication between care teams/settings, as has been acknowledged elsewhere to be a barrier to optimal diabetes care.^23^ Offering SIH‐specific training and improving discharge processes might help to improve the capacity of primary care to take care responsibility for SIH post‐discharge, easing the SIH‐related workload that was reported by the inpatient diabetes care team in the present study and elsewhere.^5^

Generally, patients and family rated highly the care and service received from the hospital, after discharge, in the form of regular monitoring phone calls from diabetes specialist nurses. This was reported to help build their confidence in managing SIH at home and to increase satisfaction. Follow‐up support is known to help build sustainable changes and adherence to diabetes management protocols,^24^ promote better glycaemic control and reduce the risk of developing diabetes‐related complications.^25^ Regular follow‐up is particularly beneficial for individuals on long‐term steroid therapy to ensure their SIH management protocol is adjusted in response to reviews of the steroid treatment.^12^

Family involvement in care was also discussed and highlighted as an area for improvement. Our participants expressed the desire for their family members to be involved throughout their treatment journey. Research shows that family support is important in maintaining good glycaemic control, particularly for newly diagnosed diabetes patients as they adapt to a new way of life and treatments^26^ and for those with multimorbidity as they navigate the complexity of multiple treatment regimens.^27^ Family members can also be a key source of instrumental support (helping with tasks such as making clinic appointments, insulin injections, preparing meals and other domestic duties) and of emotional support by providing comfort and encouragement when patients are distressed or frustrated.^28^ It is also crucial to incorporate family support (e.g. provision of diabetes care education) as part of the patient's diabetes care plan^29^ because they tend to share the burden of disease^30^ when they adapt their routines and lifestyle, such as in meal choices and timing, to accommodate the needs of loved ones with SIH.

### 4.1 Strengths and limitations

A key strength of our study is that it addresses an under‐researched subject, and yet one of increasing clinical significance with implications for individuals and care services. The idea for this study was conceived by clinicians and further informed by individuals with first‐hand experience of SIH, indicating its relevance. Inclusion of family members and members of direct care teams in our interviews adds an extra dimension to the findings and provides a holistic picture of the subject. Nevertheless, the study has several limitations, including that participants were recruited from a single hospital, and care experience may differ between hospitals and between general practices. Socio‐economic data—which could have offered deeper insight into the barriers and experiences—was not in clinical records and collected as part of this study. The sample size might be considered small; however, no new information emerged from the interviews conducted towards the end of the data collection period. To assess the representativeness of our sample, we compared the frequencies of steroid indications with those of other patients who had required input from our inpatient diabetes team during the study period and found these to be broadly similar (data not shown).

## 5 CONCLUSION AND IMPLICATIONS FOR CARE

Managing SIH out of hospital is challenging for patients, family members and clinicians. The challenge is exacerbated by the presence of multimorbidity, insufficient communication between care teams and resource limitations in primary and community care services. Our findings emphasise the importance of improving care pathways with the provision of clear, individualised care plans that should be composed before the patients leave hospital and provided in writing to the patients, their families if appropriate and primary and community care teams in a timely fashion. This underscores the importance of seven‐day inpatient diabetes specialist service provision.

Patients and their families were clear in their view that glucose monitoring technologies could be beneficial. Cloud‐based sharing of capillary blood glucose and/or continuous glucose monitoring results could help coordinate multi‐specialty, multidisciplinary and multi‐organisation care provision, particularly if the cloud platform allows real‐time written communication, alongside glucose results, between patients and healthcare professionals. Such platforms have been shown to be of value in another clinical setting, gestational diabetes, in which insulin resistance changes rapidly.^31^ Clinical trials of cloud‐based data‐sharing platforms in SIH should be conducted and priority given in their design to patient‐reported outcome measures. Furthermore, a clinical trial is justified to compare self monitored capillary blood glucose monitoring with continuous glucose monitoring for the outpatient management of SIH treated with insulin or sulfonylureas in the context of tapering steroid doses, with a focus on patient‐reported outcome measures and healthcare costs.

Offering training for managing SIH, along with the sharing of skills in insulin dose titration and resources between care teams to enable efficient monitoring of blood glucose levels, steroid treatment and facilitate medication titration, may boost the confidence of primary care providers in managing this difficult condition in community settings and thus reduce reliance on diabetes specialist care services. Finally, we call for the development of software‐based decision support tools for use by patients and/or in primary care that draw on continuous glucose monitor data to provide specific insulin dosing advice that adapts to tapering steroid doses.

## CONFLICT OF INTEREST STATEMENT

The authors have no conflict of interest to declare.

## References

[1] Trence DL . Management of patients on chronic glucocorticoid therapy: an endocrine perspective. Prim Care. 2003;30(3):593‐605. 14692203 10.1016/s0095-4543(03)00038-1

[2] Van Raalte D , Ouwens D , Diamant M . Novel insights into glucocorticoid‐mediated diabetogenic effects: towards expansion of therapeutic options? Eur J Clin Investig. 2009;39(2):81‐93. 19200161 10.1111/j.1365-2362.2008.02067.x

[3] Donihi AC , Raval D , Saul M , Korytkowski MT , DeVita MA . Prevalence and predictors of corticosteroid‐related hyperglycemia in hospitalized patients. Endocr Pract. 2006;12(4):358‐362. 16901792 10.4158/EP.12.4.358

[4] Fong AC , Cheung NW . The high incidence of steroid‐induced hyperglycaemia in hospital. Diabetes Res Clin Pract. 2013;99(3):277‐280. 23298665 10.1016/j.diabres.2012.12.023

[5] Younes YR , Stockley S , Keegan L , et al. COVID‐19 and dexamethasone‐induced hyperglycaemia: workload implications for diabetes inpatient teams. Diabet Med. 2022;39(2):e14716. 34651335 10.1111/dme.14716 PMC8646374

[6] Delfs N , Struja T , Gafner S , et al. Outcomes of hospitalized patients with glucocorticoid‐induced hyperglycemia—a retrospective analysis. J Clin Med. 2020;9(12):4079. 33348743 10.3390/jcm9124079 PMC7765857

[7] Suh S , Park MK . Glucocorticoid‐induced diabetes mellitus: an important but overlooked problem. Endocrinol Metab. 2017;32(2):180‐189. 10.3803/EnM.2017.32.2.180 PMC5503862 28555464

[8] James J , Robert A , Dhatariya K , Joint British Diabetes Societies (JBDS) for Inpatient Care . Management of Hyperglycaemia and Steroid (Glucocorticoid) Therapy: a guideline from the Joint British Diabetes Societies (JBDS) for Inpatient Care group. 2023 https://abcd.care/sites/default/files/site_uploads/JBDS_Guidelines_Current/JBDS_08_Management_of_Hyperglycaemia_and_Steroid_%28Glucocorticoid%29_Therapy_with_QR_code_January_2023.pdf 10.1111/dme.13675 30152586

[9] Melissa D , Lisa M . Semistructured interviewing in primary care research: a balance of relationship and rigour. Chin Gen Pract. 2019;22(23):2786. 10.1136/fmch-2018-000057 PMC6910737 32148704

[10] Ritchie J , Spencer L , O'Connor W . Carrying out qualitative analysis. Qual Res Pract. 2003;2003:219‐262.

[11] Wilhelmsen NC , Eriksson T . Medication adherence interventions and outcomes: an overview of systematic reviews. Eur J Hosp Pharm. 2019;26(4):187‐192. 31338165 10.1136/ejhpharm-2018-001725 PMC6613929

[12] Shah P , Kalra S , Yadav Y , et al. Management of glucocorticoid‐induced hyperglycemia. Diabetes Metab Syndr Obes. 2022;15:1577‐1588. 35637859 10.2147/DMSO.S330253 PMC9142341

[13] Hernandez CA . Integration: the experience of living with insulin dependent (type 1) diabetes. Can J Nurs Research Arch. 1996;28(4):37‐56. 9128475

[14] Audulv Å . The over time development of chronic illness self‐management patterns: a longitudinal qualitative study. BMC Public Health. 2013;13:1‐15. 23647658 10.1186/1471-2458-13-452 PMC3649883

[15] Eroglu N , Sabuncu N . The effect of education given to type 2 diabetic individuals on diabetes self‐management and self‐efficacy: randomized controlled trial. Prim Care Diabetes. 2021;15(3):451‐458. 33674221 10.1016/j.pcd.2021.02.011

[16] Mohn J , Graue M , Assmus J , et al. Self‐reported diabetes self‐management competence and support from healthcare providers in achieving autonomy are negatively associated with diabetes distress in adults with type 1 diabetes. Diabet Med. 2015;32(11):1513‐1519. 26032125 10.1111/dme.12818 PMC4744962

[17] Gallacher K , May CR , Montori VM , Mair FS . Understanding patients' experiences of treatment burden in chronic heart failure using normalization process theory. Ann Fam Med. 2011;9(3):235‐243. 21555751 10.1370/afm.1249 PMC3090432

[18] Maneze D , Weaver R , Kovai V , et al. “Some say no, some say yes”: receiving inconsistent or insufficient information from healthcare professionals and consequences for diabetes self‐management: a qualitative study in patients with type 2 diabetes. Diabetes Res Clin Pract. 2019;156:107830. 31465812 10.1016/j.diabres.2019.107830

[19] Carpenter DM , DeVellis RF , Fisher EB , DeVellis BM , Hogan SL , Jordan JM . The effect of conflicting medication information and physician support on medication adherence for chronically ill patients. Patient Educ Couns. 2010;81(2):169‐176. 20044230 10.1016/j.pec.2009.11.006 PMC2891323

[20] Andersen JD , Jensen MH , Vestergaard P , Jensen V , Hejlesen O , Hangaard S . The multidisciplinary team in diagnosing and treatment of patients with diabetes and comorbidities: a scoping review. J Multimorb Comorb. 2023;13:26335565231165966. 36968789 10.1177/26335565231165966 PMC10031602

[21] Bratcher CR , Bello E . Traditional or centralized models of diabetes care: the multidisciplinary diabetes team approach. J Fam Pract. 2011;60:S6‐S11. 22336928

[22] Kangas S , Rintala T‐M , Jaatinen P . An integrative systematic review of interprofessional education on diabetes. J Interprof Care. 2018;32(6):706‐718. 30040507 10.1080/13561820.2018.1500453

[23] Raaijmakers LG , Hamers FJ , Martens MK , Bagchus C , de Vries NK , Kremers SP . Perceived facilitators and barriers in diabetes care: a qualitative study among health care professionals in The Netherlands. BMC Fam Pract. 2013;14:1‐9. 23937325 10.1186/1471-2296-14-114 PMC3751909

[24] Campbell F , Lawton J , Rankin D , et al. Follow‐up support for effective type 1 diabetes self‐management (the FUSED model): a systematic review and meta‐ethnography of the barriers, facilitators and recommendations for sustaining self‐management skills after attending a structured education programme. BMC Health Serv Res. 2018;18:1‐24. 30482202 10.1186/s12913-018-3655-z PMC6258400

[25] Anjana RM , Shanthirani CS , Unnikrishnan R , et al. Regularity of follow‐up, glycemic burden, and risk of microvascular complications in patients with type 2 diabetes: a 9‐year follow‐up study. Acta Diabetol. 2015;52:601‐609. 25539883 10.1007/s00592-014-0701-0

[26] Shi M , Xu MY , Liu ZL , et al. Effectiveness of family involvement in newly diagnosed type 2 diabetes patients: a follow‐up study. Patient Educ Couns. 2016;99(5):776‐782. 26763869 10.1016/j.pec.2015.12.018

[27] Tong WT , Vethakkan SR , Ng CJ . Why do some people with type 2 diabetes who are using insulin have poor glycaemic control? A qualitative study. BMJ Open. 2015;5(1):e006407. 10.1136/bmjopen-2014-006407 PMC4316456 25633285

[28] Pamungkas RA , Chamroonsawasdi K , Vatanasomboon P . A systematic review: family support integrated with diabetes self‐management among uncontrolled type II diabetes mellitus patients. Behav Sci. 2017;7(3):62. 28914815 10.3390/bs7030062 PMC5618070

[29] Zhang H , Zhang Q , Luo D , et al. The effect of family‐based intervention for adults with diabetes on HbA1c and other health‐related outcomes: systematic review and meta‐analysis. J Clin Nurs. 2022;31(11–12):1488‐1501. 34888968 10.1111/jocn.16082

[30] Meiliana SW , Hakim FO , Hayati YS , Kristianingrum ND , Kartika AW , Sandi PS . The burden of family caregivers in the Care of Type 2 diabetes mellitus patients: a literature review. J Rural Community Health Nurs. 2024;2(1):40‐47.

[31] Mackillop L , Hirst JE , Bartlett KJ , et al. Comparing the efficacy of a mobile phone‐based blood glucose management system with standard clinic care in women with gestational diabetes: randomized controlled trial. JMIR Mhealth Uhealth. 2018;6(3):e9512. 10.2196/mhealth.9512 PMC5883074 29559428

